# Customer Profitability Analysis in decision-making–The roles of customer characteristics, cost structures, and strategizing

**DOI:** 10.1371/journal.pone.0296974

**Published:** 2024-05-22

**Authors:** Rainer Lueg, Dima Ilieva

**Affiliations:** 1 Institute of Management, Accounting & Finance, Leuphana University, Lüneburg, Germany; 2 Department of Business and Economics, University of Southern Denmark, Kolding, Denmark; Universidad Central de Chile, CHILE

## Abstract

**Purpose:**

This study investigates the interplay between strategic goals and calculative practices, specifically Customer Profitability Analysis (CPA). Drawing on practice-based theories, the research aims to understand how managers strategize with CPA, including the balancing of financial and strategic objectives and the interplay of institutionalized practices with individual practitioners’ actions.

**Design:**

The study uses a qualitative, revelatory, and exploratory case study approach at the sub-organizational level in a manufacturing company. The researchers compare CPA practices across six departments, guided by a phenomenological research design. Data collection methods include informal conversations, qualitative observations, written documentation, numerical evidence from the accounting system, and interviews.

**Findings:**

The study offers four novel findings to the field. First, it highlights how managers employ procedural and interactive strategizing to reframe CPA practices. The sophistication of CPA practices increases with unevenly distributed customer volume, high customer-specific, controllable overhead, customer-to-customer interaction, and service complexity. Conversely, the sophistication of cost-focused CPA practices tends to decrease with diverse strategic goals. Additionally, CPA become more effective through the utilization of non-financial information, employee empowerment, localization, and strategic alignment. Second, CPA can be adapted through integrative strategizing where managers avoid using it as a financial benchmark for strategic initiatives. Third, accountants actively seek intermediary roles to incorporate arguments from strategy and marketing to balance strategic objectives–contrary to their portrayal as myopic guardians of profitability. Fourth, the localization of CPA practices to front-line employees compensates for a lack of sophisticated CPA practices.

**Future research:**

Future research should, investigate the adaptation of calculative practices in different cultures, and industries. Exploring additional contextual factors such as uncertainty, management characteristics, and linguistic framing of practices would be beneficial. Examining the interactions in utilizing CPA practices between front-line staff and customers would shed light on their effectiveness. Lastly, investigating the role of consultants in diffusing such practices would offer valuable perspectives.

## 1 Introduction

1.1 Motivation and theoretical frame

The motivation of this study is to investigate the interplay between calculative practices and strategic goals, based on the example how managers leverage Customer Profitability Analysis (CPA) practices for pursuing strategic objectives. For this, practice-based theories have become established analytical frameworks for understanding why management practices vary across organizational contexts [1]. These theories purport that practitioners intend to pursue strategic goals of their organization by developing and legitimizing suitable practices, such as articulating targets, enforcing controls, or engaging in social interaction (*strategizing*) [2]. *Strategizing* describes how (non-top) managers try to aim their operative decision-making toward the achievement of long-term strategic goals. It is particularly interesting in relation to operative decision making with calculative practices [3]. Practices such as budgets or costing are often taken for granted as a legitimate structural context in many organizations. Thereby, these practices are the basis of procedural strategizing. They are a vehicle and sometimes even a driver of enacting strategy in daily operations [1].

### 1.2 Problem statement and working hypothesis

The problem statement of this study is the limited understanding of how practitioners perceive CPA as either a facilitator or an obstacle in achieving strategic goals. This hinders their ability to effectively strategize with CPA. Yet, the literature provides little evidence on strategizing, i.e., how calculative practices are engaged in strategy praxis [4], such as balancing financial and strategic objectives [5], the interplay of institutionalized practices with individual practitioners’ actions [1], or variations among groups [6]. In this regard, it is not only relevant for research to understand the *pattern* of how practices adapt to contexts, but to uncover *how* practitioners problematize the practice itself as either a tool or a handicap in achieving strategic objectives. This is particularly relevant for practices related to accounting. It is tempting to take their output at face value, since “*factual calculations*” lend them the legitimacy of being ‘impartial’ [7, p. 206]. However, the profitability of a product, a customer etc. can also be seen as a social construction that depends on the—possibly politically motivated—allocation of revenues and costs [4]. From this perspective, calculative practices are not impartial representations of reality [8]. Rather, they are persistent, historically embedded activities and discourse, to which powerful top management teams as well as accounting-and-finance-educated managers have better access [1, 9]. Especially in organizations with multiple strategies, the prevalence of these ostensibly ‘objective’ practices might lead to an imbalance of strategic objectives [10]. So far, only a few studies have addressed the relevance and ambivalence of calculative practices for strategizing. Practice research can still contribute more to explaining how these “*taken-for-granted*” calculative practices “*may legitimate and naturalize short-term profit-orientation*”, and how “*such practices can be resisted locally or more widely*” by reflective practitioners [6, p. 316–317]. Practices that span several fields (such as strategy, accounting, and marketing) address multiple strategies, and are hence expedient examples for such investigations [11]. Especially practices like CPA inherently confront financial with long-term strategic goals, and should cause constructive conflict among the practitioners in the departments that guard their achievement [12, 13]. It is the goal of this study to following the calls for more research on such calculative practices [5, 6, 13, 14]. It poses the research question:

*How do managers employ CPA practices to pursue strategic objectives (‘strategizing’)?*

The working hypothesis to approach this research question is that the pursuit of strategic goals and the interaction of organizational context will shape the sophistication of CPA practices.

### 1.3 Research approach

This research is a qualitative, revelatory, and exploratory case study conducted at the sub-organizational level. The study focuses on a manufacturing company and compares CPA practices across six units. Data collection methods include informal conversations, qualitative observations, written documentation, numerical evidence from the company’s accounting system, and interviews. The authors followed a phenomenological research design, involving bracketing, intuiting, analyzing, and describing, to explore managers’ use of CPA practices.

### 1.4 Novelty of this research and contributions

The study offers four novelties to this field of research. First, managers used procedural and interactive strategizing to reframe the practice suggested by the consultants. More specifically, the study suggests that the sophistication of CPA practices increases when there is unevenly distributed customer volume, high customer-specific, controllable overhead, customer-to-customer interaction, and service complexity. On the other hand, the sophistication of cost-focused CPA practices tends to decrease when there are diverse strategic goals. Furthermore, the effectiveness of CPA practices is generally enhanced through the utilization of non-financial information, employee empowerment, localization, and strategy alignment. The second novelty of this research is that CPA was not a dogmatic, financial benchmark to assess customer relationships as assumed by standard textbooks [15]. This prevented goal-mean displacement and increased the legitimacy of the practice. Even managers at lower levels in the organization kept stabilizing the practice through integrative strategizing. As a result, the authors did not observe a conflict between profitability and other strategic goals of growth and customer intimacy. Third, the study sheds light on the active involvement of accountants in intermediary roles within the organization, challenging the conventional perception of them solely as guardians of profitability. In this capacity, accountants effectively harmonize the company’s multifaceted strategic goals, particularly when financial performance data falls short of expectations. While the general manager indeed plays a pivotal role in overseeing strategic decisions, accountants prove instrumental in providing valuable insights and recommendations when addressing discrepancies in profitability outcomes. Fourth, the authors noted that a lack of sophisticated calculative practices was compensated by further localizing CPA to front line employees, which has not been documented before. This study’s insights are company-specific, but should still be comparable across many organizations in which managers use calculative practices to balance strategic objectives [3].

## 2 Theoretical background

### 2.1 Practice theory and calculative practices

Practice theory outlines how management practices are produced, reinforced, and changed in an organizational context. The theory purports that that strategy does not exist by itself, but that practitioners enact strategy through operational, situated, structurally embedded *practices* (“*strategizing*”). Practitioners combine and extend practices in episodes of *praxis* [6] and try to use them for the achievement of organizational objectives [1]. A practice consists of interconnected elements, specifically flows of activities (e.g., contemplation; discourse; routinized behavior) and structures (e.g., valuation policies/rules; infrastructures; software; budgets; general understanding of the business model, i.e., teleoaffective means-ends-relationships) [1]. The term *calculative* practice suggests that these practices are embedded in separately identifiable tools, such as algorithms, frameworks, or software applications [1, 3]. Jarzabkowski [16] classifies strategizing in more detail: *Procedural* strategizing depicts the use of calculative practices to shape strategy through diagnostic controls. This activity confers *structural legitimacy* to a practice. *Interactive* strategizing describes the continuous reinterpretation and reframing of meaning for a calculative practice. This activity lends *interactive legitimacy* to a practice by establishing normative controls of behavior. She refers to *integrative* strategizing as creating both types of legitimacy at the same time. Strategizing acknowledges that managers possess agency for “*localized exercise of judgment*” [16, p. 32] that puts the outcomes of a calculative practice in context with strategic goals.

Research in the field of accounting has followed the management researchers’ turn toward practice theories. The emergence of *strategic management accounting* (SMA) acknowledges that management accounting does not exist independently, but that it consists of calculative practices that evolve to serve strategic ends [5]. As a consequence, new calculative practices emerged that specifically aim at implementing strategies, such as customer accounting, the Balanced Scorecard, or Value-based Management [11, 17]. This development in calculative practices carries implications for the field of strategy. Vaara and Whittington [6, p. 316] denote that “*management accounting systems […] may become “obligatory passage points” in strategy-making*”.

### 2.2 Calculative practices: Empirical evidence in case studies

So far, several case studies have used practice theories to demonstrate how actors use calculative practices for strategizing: Jørgensen and Messner [18, p. 184] assess accounting practices in new product development (NPD). NPD is subject to uncertainty, limited calculability, long periods between decisions and observed outcomes, and diverse demands of various stakeholders. The authors conclude that actors strategized by going beyond the accounting information and “*mobilising different strategic objectives to which these practices are supposed to contribute*”. Perna et al. [19] also investigate NPD. They suggest that the monetization of the NPD process serves five very different purposes beyond the mere measurement of profitability: financing NPD; valuing the resources employed; enabling; as well as blocking possible development paths; and finally, distributing the value created from NPD. Wouters and Kirchberger [8] illustrate that calculations of customer value are not merely neutral representation of profitability, but reciprocally frame managers’ perceptions of customers to a degree that the case companies adjusted their offerings. Ezzamel and Willmott [7] suggest that managers saw accounting information as the most faithful representation of abstract strategic concepts. Therefore, accounting information became significant in the strategic discourse. The new roles of calculative practices resulted in a change of the identities of accountants, which turned from bookkeepers to customer-focused management advisors. Seal and Mattimoe [12] illustrate how three case companies in the hospitality industry control their strategies. Accounting practices initiated discussions among organizational units and helped in adjusting strategies to environmental changes. Specifically drawing on the conflict between the finance and the marketing functions, accountants assumed various roles such as custodians, or watchdogs of organizational objectives. Sundquist et al. [20] analyze three settings of make-or-buy decisions. They highlight that many relevant aspects to such a decision are hard to quantify, and that the design of make-or-buy practices needs to be customized to the setting in which they are used. Keränen and Liozu [21] investigate how calculative practices become implemented. They propose four role configurations for implementers (“*value champions*”) and explain the rationales and positive consequences for each. Fish et al. [22] present a complementary case to the former. They explain how a CPA adoption failed due to managers that use power over argument, reject subjective judgments of others, but then prefer their own intuition to data-driven analyses. In the same vein, the case study of van der Steen and Tillema [23] illustrates how overly ridged calculative accounting practices can prevent effective use of lean manufacturing, leaving the organization with a fragmented implementation. Nielsen et al. [24] investigate how calculable practices influence strategic outsourcing decisions. The authors find that accounting information (such as customer profitability) served as an analytical decision algorithm and lent vetoing power over future strategic options. Alternatively, accounting can be one of many information sources for building discourse, without any vetoing power. The latter allowed actors to reach higher levels of strategizing. Balboni and Terho [25] show how companies can measure and manage future customer value potentials based on realized sales and non-statistical information from the sales force. They show how this approach reaches beyond internal, historical data, and accounts for customer portfolio dynamics in B2B settings. Güldenpfenning et al.’s [13] case study explains how management control systems interact in performance improvement programs, and structure them in the three mechanisms of communication, internalization, and socialization of practices. Whittle and Mueller [4] illustrate how future strategy was benchmarked against accounting information as a forceful “*obligatory point of passage*” instead of one among many information sources. In response, actors used political tactics manipulate or circumvent accounting benchmarks.

### 2.3 Classifications of Customer Profitability Analysis

CPA is a calculative practice that can be used for decision making [11, 17]. In its most basic form, CPA estimates the profits (gross margin as revenue minus direct cost) from customer transactions during a period [15]. CPA helps managers allocate costs to individual or segmented customers [15]. Thereby, CPA acknowledges that revenue and resource consumption do not equally spread across customers, even if the latter purchase the same product.

The extensive literature review of Matsuoka [11] still sees ample opportunities for research, specifically on understanding the value creation processes such as CPA [15]. One major CPA research stream is normative and offers prescriptions on cost measurement and allocations. Another major stream of research investigates the performance consequences of CPA. Yet, there are few examples from companies of how CPA-practices are generally used for strategizing [5], and in which contexts they are more sophisticated [3, 17]. The literature offers several options based on which CPA-practices can be structured, e.g., based on *strategic success factors* [3], or arithmetic *accuracy*, which improves with the number of cost drivers and cost pools. The authors categorize CPA-practices by their level of *hierarchical integration* from marginal toward full costing. This appears to be the dominant theoretical perspective on CPA [15], and also concurs with the understanding of CPA that the practitioners of this study have. The authors classify CPA-practices from pragmatic (CPA-type I) to sophisticated (CPA-type III).

#### 2.3.1 CPA-type I: Marginal costing

CPA-type I uses only financial information for assessing customer profitability. It deducts the direct costs of the customer relationship from the revenue incurred by the customer. The resulting gross margin ignores the customer-specific overhead costs. CPA-type I can support strategizing when managers discuss which course of action to take in order to maintain/improve customer profitability (i.e., *Activity-based Management*: ABM) [15]. For CPA-type I, ABM could include improving customer profitability through adjusting internal operations to customer needs (such as smaller batch sizes), and discussing contracts, pricing, and discount policies [15]. These activities imminently link to revenues and direct costs, but would involve complementary, non-financial information related to operations, legal, or strategic positioning.

#### 2.3.2 CPA-type II: Additional non-financial information

Many costs in a customer relationship do not vary with volume. Instead, they mostly link to non-financial drivers (i.e., operational activities) that consume already committed resources [14]. Hence, two customers who incur the same direct costs are probably not equally profitable, if the levels of service provided to them differ. CPA-type II extends CPA-type I by including such non-financial complexity drivers. Managers can use the information of how many activities have been performed to initiate ABM, even without knowing the exact cost of an individual activity. Examples of complexity drivers include the degree of customization, order size, predictability of orders, provision of service and advice, transportation, inventory, bargaining power of customers, shared cultural understanding of conducting business, and customer satisfaction/ loyalty/ willingness to recommend [15].

#### 2.3.3 CPA-type III: Allocation of customer-specific overhead costs

CPA-type III is a CPA-type II that additionally allocates customer-specific overhead costs and hence estimates profit margins [14]. Managers must gain a detailed understanding of the cause-and-effect relationships that determine how serving complex customers at the operative level incurs indirect costs. As a result, the number of cost pools and cost drivers is highest for CPA-type III [26]. CPA-type III allows managers a well-directed use of ABM, such as temporarily exempting new/strategically important customers from being profitable; convincing marginally profitable customers to carry over more business; or deferring customers that have little prospect to add value in the long term. CPA-practices also include that (non)-financial data are elaborated in ongoing customer profiles, or in periodic CPA reports.

## 3 Research design

### 3.1 Case study methodology

The authors conducted a qualitative, revelatory and exploratory case study at the sub-organizational level for three reasons. First, it provides a holistic perspective on practices and their application [3]. The authors compare CPA-practices at six units of a manufacturing division (the ‘company’). This single-company-case-approach lowers confounding effects of a cross-sectional sample, while still being able to explain variance across the units [1]. Since advanced CPA-practices are most likely to be found in the manufacturing industry [14], the authors chose a manufacturer. Second, it gave the authors the opportunity to constantly augment their understanding of practices by corroborating the fit of the data with theoretical perspectives. Third, investigating calculative practices matches well with a case study, especially since it provides *“[…] richer insight into explaining why*, *and under what circumstances*, *some organizations adopt simplistic systems and others do not”* [26, p. 421]. Relevant CPA-related information is not fully quantifiable, not publicly available, and warrants detailed contextual insights for interpretations [11, 17]. Therefore, the approach is qualitative and based on multiple sources of data. The study uses anonymized data and, therefore, did not require ethics committee consent from Aarhus University and Leuphana University, where the study was conducted. Informed consent was secured and obtained verbally from all participants for their inclusion in the study.

### 3.2 The case company

The authors chose this company with its specific divisions due to the five reasons of its openness to diversity (and hence practice variation); the existence of multiple, possibly conflicting strategic goals; the high managerial discretion to apply or reject CPA at the division level; the complex matrix structure that required the mindful balancing of operational objectives; and the elimination of confounding effects across companies.

#### 3.2.1 Strategic objectives and ownership

The parent of the case company is a listed corporation with a long history of pioneering manufacturing. It had won several prizes for the diversity of its workforce and valued its staff’s opinion as an antecedent to innovation and customer intimacy. This openness to diversity provides a fertile soil for variations in management practices [1], which was the first reason why this company was chosen for this research. The parent corporation’s primary mission was to create long-term shareholder value, subject to diligent corporate governance. Hence, it followed multiple strategies that are of particular interest to this field of research [1], which was the second reason for choosing this company. The two objectives *profitability* and *growth* add up to *profitable growth*. These objectives reflected the financial interests of shareholders who were mainly institutional investors from the U.K. and the U.S.. Employees equally aligned with this strategic direction, since approximately one third of them owned shares in the corporation. The third strategic priority of the parent corporation was *customer intimacy*. This gave an indication of the corporate mindset, which was both technology-based and strongly orientated towards marketing technologies.

#### 3.2.2 Organizational structure

The parent corporation coordinated semi-autonomous divisions that corresponded to its main product families. It allowed these divisions’ headquarters to delegate decision making power to their SBUs. The research focused on the largest division that comprises most of both corporate sales and personnel. The authors refer to this division as ‘the company’ or ‘CIG’ (for consumer and industrial goods). CIG operated predominantly in the business-to-business (B2B) market and served most of its end consumers via distributers. Its B2B-customers differ substantially in volume, product mix, and level of service complexity. CIG had discretion on the pricing of products and services independently of its parent corporation, which constituted the third reason to investigate this company. The SBUs mirrored the product groups of CIG. They ranged from products for end customers to industrial products. All SBUs were structured as matrices that combine the vertical management hierarchies with horizontal functions and allowed CIG to simultaneously focus on several dimensions, i.e., on customers, products, regions, and functional excellence. This need for balancing diverse objectives was the fourth reason why this company was suitable for this kind of research. Fifth, the authors chose a single company to avoid confounding effects: a single company strengthens the argument that contextual factors are strong enough to cause different CPA-sophistication even among units of the same company. At the same time, a single company case eliminates confounding effects of a cross-sectional sample. The authors refer to the six units as *SBU1* to *SBU5*, and *CCU* (central controlling unit of CIG).

### 3.3 Data sources

We followed CIG over a total of 12 months, during which time the authors had an office in CIG’s headquarters. The authors conducted a CPA project during the last seven months and avoided intervention with the CPA-practices. CIG’s top management granted the authors access due to its interest in knowledge exchange with academia. It cooperated with several university-based research teams like the authors’ to facilitate academic, customer-related research as an independent complement to CIG’s consultants. CIG let the authors choose their research focus and was supportive of their requests for data access. The main contact was the Senior Vice President of supply chain, operations, and R&D controlling, who reported directly to the CFO of CIG. Four of the SBU-headquarters were located nearby, which facilitated personal contact with most managers involved.

The primary sources of data are informal conversation, qualitative observations, written documentation, and numerical evidence drawn from the company’s accounting system. The authors reviewed the CPA-reports of all units as well as guidelines and manuals for their regular update. As expected, the authors observed that that the units with more sophisticated CPA-practices exhibited more formalized CPA-manuals and CPA-reports. The authors had access to the ERP-systems and customized spreadsheets. Additionally, the authors could perform analyses with customer data to support their research (cf. section 4.3).

We corroborated these data with interviews. Since the authors had at least weekly interaction and informal conversations with the owners of CPA, the authors developed an emic understanding of their practices. The authors scheduled official interviews towards the middle of their project to clarify issues raised from their observations and interactions with managers. The authors did not predetermine the number of formal interviews, but adjusted their research to considerations such as saturation (due to redundant information) or the time constraints of their participants. The authors interviewed 14 managers from CIG and one consultant during a two-month period in 14 formal sessions. With the exception of one phone interview, the authors conducted all of the interviews in person. The authors prepared a protocol of open-ended questions for discussion and provided these to the interviewees in advance (please see the appendix for the development and list of questions). The questions guided the conversations in the areas of CPA as a calculative practice, and the context of the embedded practice. The semi-structured character of the interviews allowed the authors to clarify how CPA links to decision-making, reporting, and mindsets of the units’ managers. The authors adjusted their basic list of questions to the interviewees based on their responsibilities, but the authors always covered all main topics. Topics included, for example, definition of customer profitability; characteristics of the CPA currently used (e.g. content/sophistication/quality of information); CPA-owners; CPA-users; CPA-usage (e.g. decision making, performance evaluation, monitoring); initial expectations upon CPA-adoption; further applications and requirements of CPA-output (e.g. further areas of interest and future developments); CPA-challenges and constraints; additional information needs and alternative sources of information; customer characteristics; business model; controllability. All interviews were transcribed and approved by the interviewees (cf. Table 1). The authors used informal meetings and further phone calls to address additional questions toward the end of their research project. The authors are confident that their emic understanding of CIG’s CPA-practices can inform this case, of which the complementary interviews only form a minor part.

**Table 1:** Interviewees.

#	Position of interviewee	Level*	Unit**	Function	CPA ownership	Length of formal interview
1	Senior Vice President	3	CCU	Controlling & Supply Chains	Yes	Ongoing
2	Business Unit Controller	5	CCU	Controlling & Supply Chains	Yes	45 min
3	Business Unit Controller	5	CCU	Controlling & Supply Chains	Indirectly	Ongoing
4	Senior Vice President	3	CCU	Controlling (regional)	No	40 min
5	Director	4	SBU1	Controlling & Finance	Yes	50 min
6	Regional Key Account Manager	6	SBU1	Marketing	No	45 min
7	Director	4	SBU2	Controlling & Finance	Yes	50 min
8	Business Unit Controller	5	SBU3	Controlling & Finance	Yes	50 min
9+10	Directors	4	SBU4	Controlling & Finance	Yes	70 min
11	Business Unit Controller	5	SBU4	Controlling & Finance	Indirectly	30 min
12	Business Unit Controller	5	SBU4	Controlling & Finance	Yes	45 min
13	Business Unit Controller	5	SBU4	Controlling & Strategy	Yes	70 min
14	Director	4	SBU5	Controlling & Supply Chains	Yes	60 min
15	External consultant	-	external	Consulting (external)	Indirectly	50 min

* Level: 1 = Member Executive Board (Corporate); 2 = Executive Vice President (Divisional board); 3 = Senior Vice President; 4 = Director; 5 = Business Unit Controller/Manager; 6 = Key Account Manager.

** Unit: CCU = Central Controlling Unit; SBU1-5 = Strategic Business Unit 1–5.

### 3.4 Data evaluation (phenomenological design)

To ensure a rigorous exploration of the managers’ experiences and perceptions regarding the implementation of CPA practices, the authors adopted a phenomenological research design consisting of four key steps: bracketing, intuiting, analyzing, and describing. In the *bracketing* phase, the authors consciously set aside their preconceived notions, for instance, that a highly profitable customer (according to CPA) is always preferable to a less profitable customer. This allowed the authors to delve into the managers’ accounts without imposing their own interpretations.

During the *intuiting* phase, the authors engaged in a process contemplation, seeking to grasp the underlying meanings and essences inherent in the participants’ lived experiences. For this first cycle of *structural* coding [27] the authors used the two constructs of ‘profitable growth’ and ‘customer intimacy’ that the authors derived from the analysis of the corporate strategy. This helped the authors in categorizing managers’ decision-making processes with CPA according to the corporate strategy. As a result, the authors also structured their findings section based on the strategic goals of profitable growth (section 4.2) and customer intimacy (section 4.3).

The subsequent *analyzing* phase of *re-coding* [27] involved systematically organizing, categorizing, and synthesizing the data to identify common themes, patterns, and insights that emerged from the managers’ narratives. The authors first catalogued decision-making processes for each strategic goal to systematize the CPA and make it comparable across the units. Common themes emerged that related to traits of overheads (section 4.2.1), future orientation (4.2.2), customer volume (4.3.1), customer-to-customer interaction (4.3.2), customer service complexity (4.3.3), and balancing the two main strategic goals of profitable growth with customer intimacy (4.3.4). Second, the authors created cross-case displays to facilitate comparisons across units (see any figure or table in this article).

**Fig 1:**
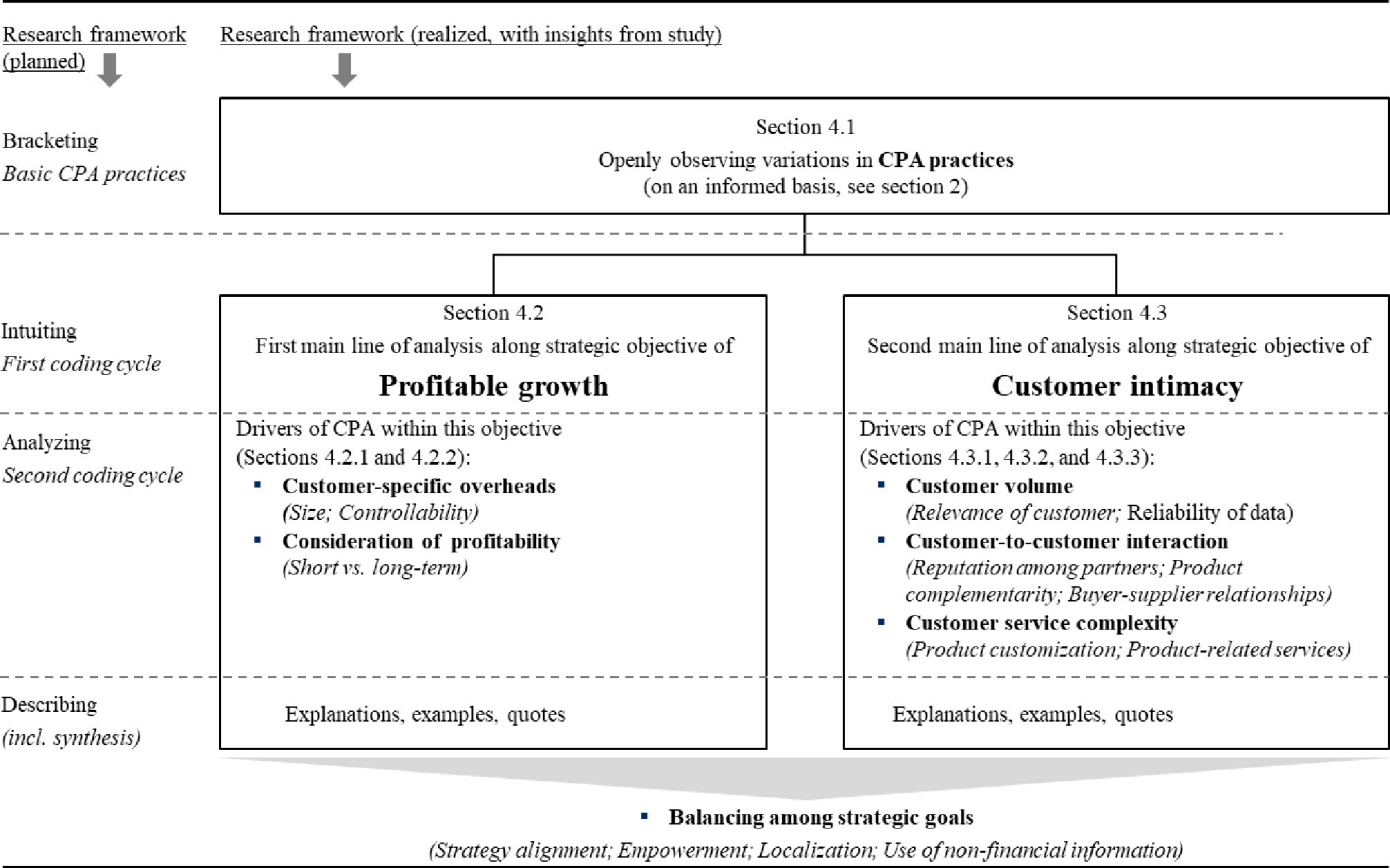
Phenomenological design and structure of findings section.

Finally, in the *describing* phase, the authors articulated a comprehensive and vivid portrayal of the managers’ experiences with CPA practices, capturing the nuances, complexities, and contextual factors that influenced their perceptions and decision-making processes. By following this phenomenological research design, the authors aimed to provide an in-depth understanding of the subjective realities and subjective meaning attributed to the implementation of CPA practices within the organization. The design of this study is depicted on the left-hand side of Fig 1. For orientation purposes, the right-hand side of Fig 1 already depicts the realized post-study design.

## 4 Findings on CPA-practices for strategizing

While the strategic objectives of CIG were universal (*profitable growth* and *customer intimacy*), the six units dealt with particular strategic contexts in terms of cost structures and opportunities for pricing (relating to the objective of *profitability*), markets (*growth*), and *customer* characteristics. Therefore, the units strategized based on different CPA-practices. The authors first describe the basic sophistications of CPA-practices. The authors continue by explaining how strategic objectives shaped CPA, and how managers balanced the multiple strategic objectives. The authors structure their findings by strategic objectives.

### 4.1 Variations in CPA-practices across the six units

CIG wanted to follow the suggestions of a consulting firm to implement CPA-type III across the division. The company opted for a decentralized implementation strategy [(specifically for value-based marketing, cf. 21]. Corporate top management localized the practice to the divisions, who further localized it to their units. Once the consultants had left, CIG’s units considered implementing CPA-type III by running pilot tests. Eventually, SBU1, SBU2, SBU3, and CCU decided in favor of a ‘pragmatic’ implementation that reframed the practice. Managers argued that the complexity of CPA-type III outweighed the improvements in decision usefulness:

*“In the past*, *we have tried to implement a concept similar to the profit margin but have… ‘failed’? Well*, *actually not*. *It would be more appropriate to say that the concept just didn’t work so well here*.*” (Director Controlling*, *SBU2)*

CIG’s top management interest in this development initiated their research project. SBU1, SBU2, and CCU used gross margins to assess the profitability of their largest 10–100 customers. Ad hoc analyses of extreme cases included a more detailed assessment of profitability for individual top accounts. In addition, CCU performed a monthly overdue analysis and initiated ABM if appropriate.

SBU3 implemented CPA-type II which extended CPA-type I by including non-financial information, particularly order patterns. This information did not support a numerical allocation of customer-specific overhead costs. Yet, the knowledge of these patterns enabled managers to initiate ABM, e.g., to improve order predictability.

SBU4 applied CPA-type III that allocated customer-specific overhead costs to calculate profit margins on customers. The cost drivers were related to differently priced employee hours [time-driven ABC: 15]. CPA covered the whole customer base rather than just top accounts, clustering smaller customers into segments. SBU4 accounted for non-financial drivers of profitability, especially customization requirements, customers’ business focus (cost saving vs. innovation), and sporadically the key customers’ willingness to recommend (‘word of mouth’). To accommodate the shortsighted and risk-neutral view of CPA, SBU4 was also contemplating to implement CLV analysis to assess the future profitability of customers, but had not finished this process. Due to the specificity of their products, managers regarded order patterns as hardly manageable, and did not track them.

SBU5 also allocated customer-specific overheads (CPA-type III). As in SBU4, this added to the comprehensive customer profiles, which stemmed from hundreds of customer interviews. Additionally, CPA evaluated customization requirements and order patterns. SBU5 clustered smaller customers in groups. SBU4 and SBU5 updated much of their information monthly, as opposed to the quarterly/annual updates of the other units.

All six units use ERP modules to record and report revenues and costs. SBU3, SBU4, and SBU5 stored additional non-financial information in spreadsheet software. Table 2 summarizes the sophistication of the calculative practice at the units.

**Table 2:** Classification of units according to CPA-type.

CPA-type	Cost drivers	CCU	SBU1*	SBU2*	SBU3*	SBU4	SBU5
**1:** Gross margin 1	*Net external sales (after discounts)*	O	O	O	O	O	O
	*Gross margin*	O	O	O	O	O	O
**2:** Gross margin 2	*Order pattern (e*.*g*., *sizes*, *frequency*, *delivery terms)*	-	-	-	O	-	O
	*Business focus of customer*	-	-	-	-	O	-
	*Customization requirements*	-	-	-	-	O	O
	*Future profitability*	-	-	-	-	O	-
	*Willingness to recommend (word-of-mouth)*	-	-	-	-	O	-
	*Customer satisfaction* **	-	-	-	-	-	-
	*Customer bargaining power* **	-	-	-	-	-	-
	*Customer proximity* **	-	-	-	-	-	-
**3:** Profit margin	*Several (see e*.*g*., *above) until profit margin can be assessed*	-	-	-	-	O	O
**CPA-type**		**I**	**I**	**I**	**II**	**III**	**III**

" * Earlier attempts to implement CPA-type 3 (profit margin).

** Discussed but not implemented at the unit level"

### 4.2 Decision-making with CPA for profitable growth

Decision-making with CPA-practices adjusted to strategic objectives (*strategizing*). *Profitable growth* linked to the size and controllability of customer-specific overhead costs (4.2.1), and balanced current with future profitability (4.2.2).

#### 4.2.1 Size and controllability of customer-specific overhead costs

Precursors of customer profitability, like Atkinson, Kaplan [15], argue that cost structures have shifted toward a higher relative amount of overheads during the 20^th^ century. The authors propose that higher relative overhead costs should lead to the deployment of sophisticated customer accounting. The authors found partial evidence for this thinking at CIG.

**Fig 2:**
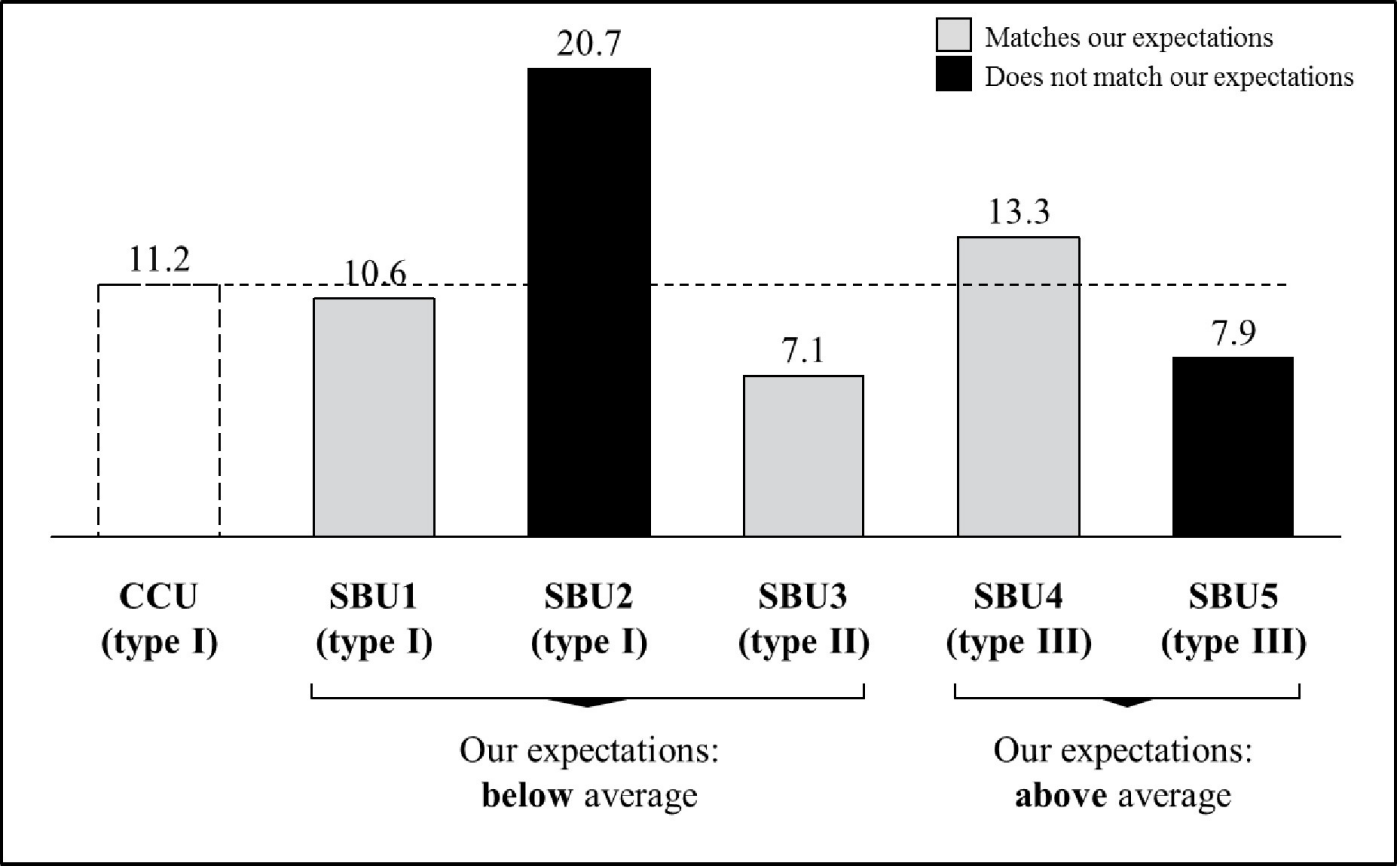
Customer specific overhead costs* (in % of sales).

CIG had consistent definitions for customer-specific overhead costs, including cost of the field sales force, order fulfillment, regional and central supply chain offices, marketing, (technical) service, and key account management. CIG treated customer-specific overhead as fixed in the short term, but saw them as avoidable in the mid-term. CIG monitored such ‘avoidable fixed costs’ and initiated ABM if some customer relationships fell short of achieving long-term profitability. The authors considered customer-related overheads as ‘relatively high’ if they exceeded CIG’s average (cf. Fig 2).

SBU4 and SBU5 practiced the most sophisticated CPA-type III that allocates all customer-specific overhead costs. Based on Atkinson, Kaplan [15], the authors did not expect that SBU5 had such sophisticated CPA-practices, given their low overhead costs. Likewise, the authors were surprised that SBU2 had the highest relative customer-specific overhead costs but the lowest CPA-sophistication. A manager reasoned:

*“Basically*, *about 83% of sales come from customers where it takes too much effort to first calculate and then allocate overhead costs […]*. *It is hard to acquire such data in a manner that is both reliable and doesn’t consume too much time*.*” (Director Controlling*, *SBU2)*

Similarly, managers in SBU5 accepted this ‘misfit’ as they saw customer characteristics as more determining for the optimal sophistication of CPA-practices than cost structures.

As to the controllability of overhead costs, SBU1 and CCU were active in an end consumer-oriented business. Standardization of products and services was high, which lowered the controllability of costs. Any change in overheads affected the profit margins of all customers in a given unit equally. Hence, managers in these units did not see much sense in tracking overheads:

*“Sometimes it is*, *however*, *impossible or difficult to change their ordering behavior*, *e*.*g*., *if the whole market behaves like this*, *or if the customer is really big and has a lot of influence*. *It’s not only an issue if we can detect the order pattern but also if we are able to do something about it*.*” (Regional Key Account Manager*, *SBU1)*

SBU3-5 employed CPA-practices that were more sophisticated. They were active in the industrial-oriented business, where overheads are scalable to customer demands. Again, SBU2 did not fit their expected pattern. CPA-practices were not very sophisticated even though the unit was in the industrial-oriented business where customer-specific overheads were mainly controllable. As quoted above, managers at SBU2 argued that customers characteristics were determining for CPA-practices, and cost structures were a secondary criterion. From these findings, the general propositions can be derived:

Proposition 1a: Relatively high customer-specific overhead costs are positively associated with the sophistication of CPA practices.

Proposition 1b: Controllability of customer-specific overhead costs are positively associated with the sophistication of CPA practices.

#### 4.2.2 Balancing past profitability and profitable growth

Managers showed awareness that CPA did not contain information on future profitability. The authors observed that managers did not see CPA as the unquestioned yardstick that evaluated customer relationships. Instead, they simultaneously considered achieving the objective of *profitable growth*. Managers mainly used CPA for *procedural* strategizing [3] that involved diagnostic controls, such as variance analyses. Especially managers from units with less sophisticated CPA-practices alerted that the numerical analyses should not be overemphasized. They highlighted that gross margins did not contain any information relevant for future profitability or attaining strategic goals. Even the more sophisticated SBU4 and SBU5 acknowledged that many allocations were arbitrary, and that they needed to strategize beyond the calculative practice to achieve strategic objectives:

*“We are very careful about what we include in the overhead costs*. *But still*, *it involves a lot of approximation and guessing when allocating these costs to customers*.*” (Business Unit Controller*, *SBU 4)*

*“We did not really base any decision on changing the portfolio on this [CPA]*. *Monitoring yes*, *but not more*. *Because we think it is normal to have a portfolio where some customers are below average*. *And that is OK*, *for the purposes of scale and so on*. *Also it is very dangerous to base decision only on this [CPA]*. *We need different types of information to be able to make such drastic changes*.*” (Director Controlling*, *SBU5)*

The incomplete information offered by this calculative practice was also the reason why all managers opposed the idea of the consulting firm to trigger generic actions based on CPA, such as refusing service to less profitable customers:

*“If it was implemented*, *this would have meant to transform the whole organization according to this new logic and new method of calculation*. *[…] Suddenly*, *some actually profitable customers would be told: ‘We are sorry; we no longer provide this service to you*, *because you are not amongst our most profitable customers’*… *and so on*. *Should we cut customers that we have served for many years only based on one CPA? How do we justify this to top management?” (Senior Vice President Controlling*, *CCU)*

From these findings, the general proposition can be derived:

Proposition 2: Relatively diverse strategic goals are negatively associated with the sophistication of CPA practices.

### 4.3 Decision-making with CPA for customer intimacy

Decision-making with CPA for *customer intimacy* related to different customer characteristics across units, and the counterbalance of *profitability* and *growth*.

#### 4.3.1 Customer volume

**Fig 3:**
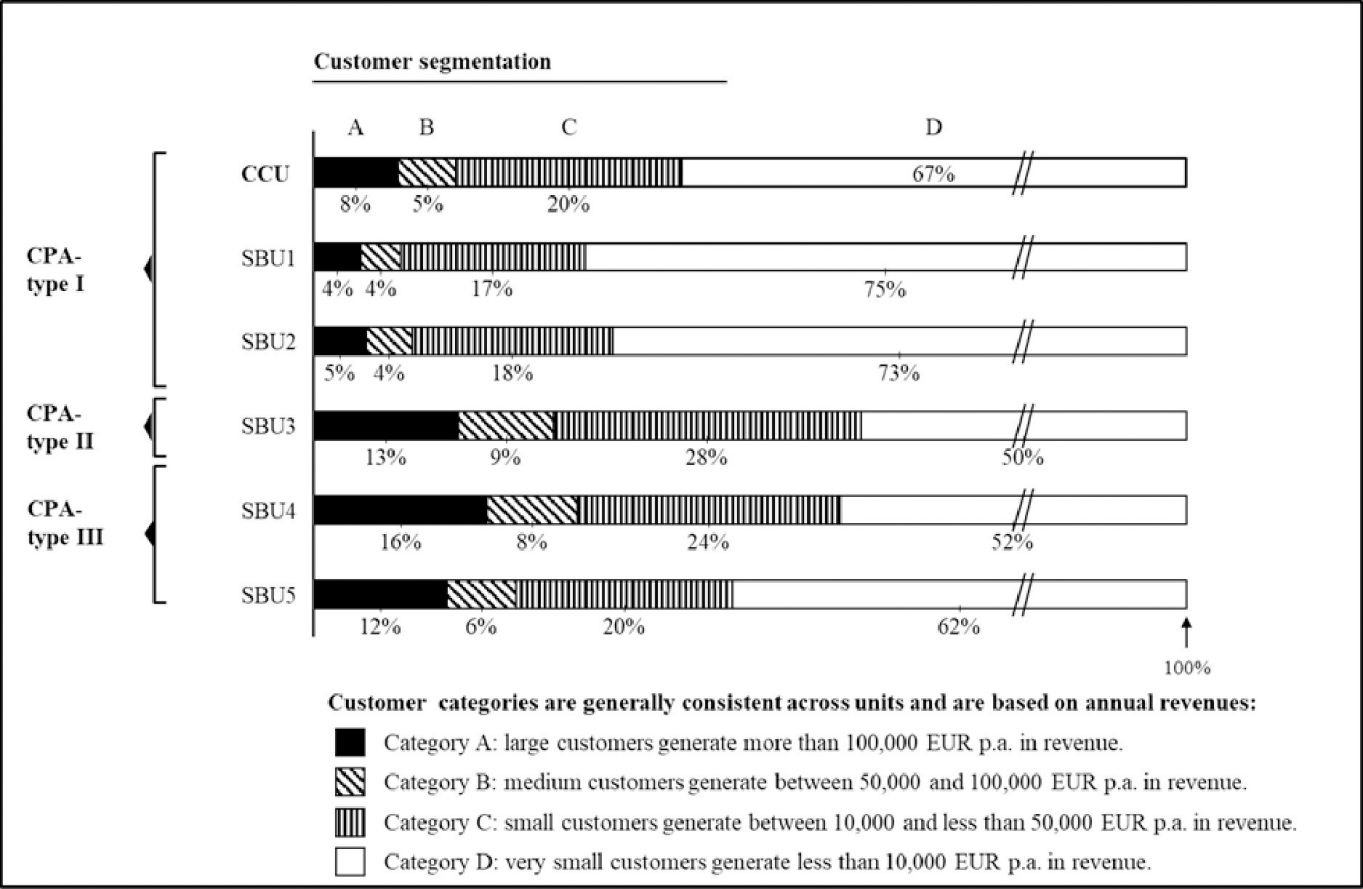
Customer segmentation (in %).

**Fig 4:**
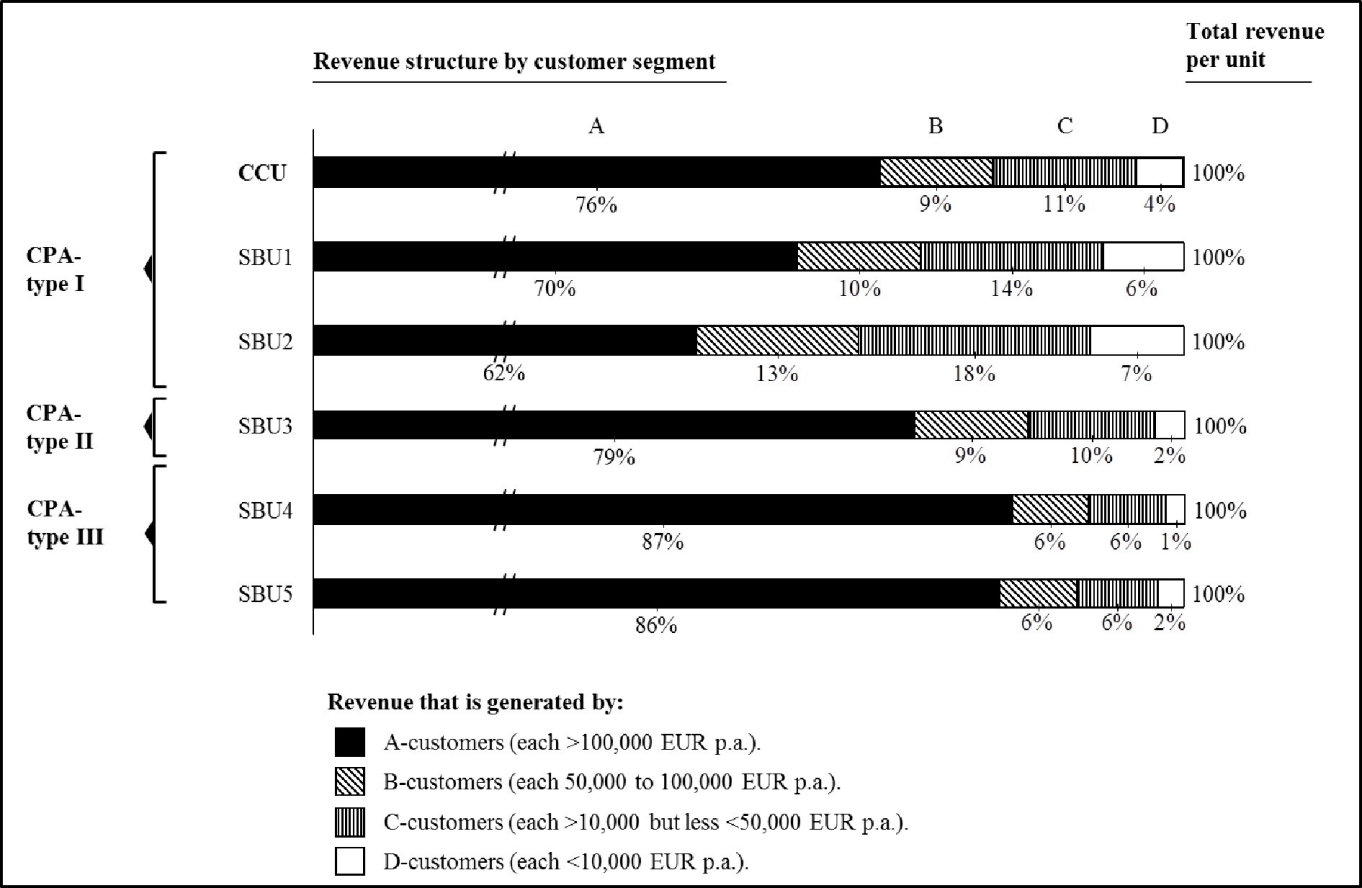
Revenue structure by customer segment (in %).

The extant literature prompts that CPA is most conducive for managing customer portfolios that contain relatively large customers by sales volume. This is because the high absolute positive/negative contribution margins make the largest customers either the most or least profitable in a portfolio [15]. CIG classified customers from A to D based on annual revenue. The authors analyzed the relative amount of A to D customers in each unit (Fig 3) as well as the revenue structures they created (Fig 4):

SBU3-5 had relatively many large customers in their portfolio, and correspondingly employed sophisticated CPA-practices. SBU1 and SBU2 had fewer large but many small customers, and they practiced only CPA-type I. Managers across units indicated that sophisticated CPA-practices appeared most sensible if the large customers generated a relatively big share of revenues. At the same time, the authors realized that reliable data for CPA was often not available if the customer portfolio contained many small customers. This was especially true for non-financial, strategic information. The CPA-type I users explained to the authors that atomized customer structures quickly revealed the limits of calculative practices:

*“We know which archetypes of customers buy our products*, *but […] their behavior is very individual*. *The customer groups are mostly so small that it is practically impossible and very impractical to be looking into each single one of them*. *[…]*. *One problem with this analysis is that the data might be a little unreliable*.*” (Director Controlling*, *SBU1)*

From these findings, the general proposition can be derived:

Proposition 3: Unevenly distributed customer volume (i.e., presence of notably large customers) are positively associated with the sophistication of CPA practices.

#### 4.3.2 Customer-to-customer interaction

SBU4 and SBU5 managers had high customer-to-customer interaction. It was important that their customers were treated the same way (e.g., in terms of discounts) if they communicated with each other. Managers saw validity problems in having a single definition of this customer-to-customer interaction. Yet, this information was too crucial to ignore it. Based on practices beyond the calculative CPA (e.g., customer profiles, conversations, and observations), the authors identified several complimentary approaches to assess customer-to-customer interaction: First, managers relied on their knowledge of partnerships between customers. A second indicator was if customers produced complementary products that an end consumer would use in combination. Third, certain characteristics of customer products indicated possible buyer-supplier relationships. If the products were subject to further processing down the industry value chain, they attributed a higher probability of customer-to-customer interaction. Such strategic, non-financial KPIs were hard to translate into customer profitability. Yet, they contained strategically relevant information. Therefore, managers found it acceptable that even fuzzy knowledge of customer-to-customer interaction could overrule an unfavorable CPA-assessment:

*“However*, *rebalancing our portfolio is tricky even if we have the necessary information to do it*. *We cannot simply accept what the CPA says and cut out a chunk from our portfolio*. *[…]*. *For example*, *we might find out that a small customer is unprofitable and decide to get rid of him*. *But he can be a key supplier of one of our big*, *important customers*. *[So] by cutting out this one*, *we might actively force other customers to end their business relations with us*.*” (Business Unit Controller*, *SBU4)*

It is remarkable that SBU4 and SBU5 exhibited this caution, given that their CPA-practices were the most sophisticated. Managers understood that a lack of strategic information in CPA was a major shortcoming and that only complementary strategizing avoided goal-mean-displacement. From these findings, the general proposition can be derived:

Proposition 4: Relatively high customer-to-customer interaction is positively associated with the sophistication of CPA practices.

#### 4.3.3 Customer service complexity

Holm et al. [14] reason that the sophistication of CPA-practices can be related to *customer service complexity*, i.e., the variation in service needs that trigger the number and duration of customer-related activities. At CIG, customer service complexity comprised product customization and product-related services. The units with more sophisticated CPA-practices (SBU3, SBU4, and SBU5) supplied products that needed to be specifically fitted—or even newly developed—for their customers’ needs. Therefore, managers constantly invested in exploring and profiling customers to understand and track the related overhead costs.

*“After we generate the necessary financial information*, *we also want to know some other ‘qualitative’ factors: what is the complexity of the customer? For example*, *the number of people which service them*. *Or the delivery terms; if we deliver to only one plant or if the customer has five different plants and so on*. *[…] Our employees go there and work with the customer*. *Sometimes even after the product is in use*, *they ask us to remain present there for monitoring*, *for support*, *for service*.*” (Director Controlling*, *SBU4)*

SBU4 was a particularly prominent in terms of customer complexity. The unit did not push products into the market. Instead, managers used non-financial accounting information on customers as a source of innovation and adjusted, customized or upgraded recent technologies to generate fit with customer needs [similar: 19, 24]. Thereby, the sophisticated CPA-practices went beyond financial information and were mobilized for *interactive* strategizing [3, 25]. This eventually led SBU4 to adjust its business model:

*“Basically we start by this so called ‘audit of the customer’*. *We go to the customer and start asking*. *We want to understand their business*, *what they need*, *and expect*. *Then we go to our lab and try to make that*. *Usually*, *we should try to use an existing application or product*, *but often we are actually trying to invent new products*. *Then we start testing*. *First we perform small scale tests in the lab*. *Then we go to the customer and run the tests there*. *[…] It is one thing to sample*, *but it is totally different if we have to produce large quantities*. *There are a lot of trials and testing on site with the customer*.*” (Director Controlling*, *SBU4)*

Contrary, managers in SBU1 and SBU2 perceived customer expectations as an exogenous factor. The units followed a push-approach in sales and a resource-based approach to innovation that focused on raising brand awareness through pioneer technologies. The involvement of the customer was rather one-directional and only included procedural strategizing. Managers saw limited opportunities for product customization or additional services. This made qualitative customer information or the allocation of overheads less relevant:

*“We don’t approach the business by looking at customers*, *mainly because of their fragmentation*. *[…]*. *Instead*, *we focus on products and technologies*. *Our starting point is a target price*. *Then we look into product segments*, *then into technologies*, *then into our latest innovations*. *Only then we—sometimes—look at the specific customer groups of each product*.*” (Director Controlling*, *SBU2)*

This statement shows that the fit CPA-practices depended on several customer characteristics that might conflict: the interviewee argued in favor of low CPA-sophistication due to low service complexity. Before, he raised the concern that low customer volume would have prevented CPA-practices that are more sophisticated, even if service complexity would have been high. The authors observed a similar example where the setting was opposite: even though a customer had a large volume, sophisticated CPA-practices were not necessary, as service complexity was low.

*“For example*, *we have [global discounter]*, *where the contribution margin is very low*, *but so are the costs of servicing them*. *They buy the standard product in the standard way and require the standard support and service*.*” (Regional Key Account Manager*, *SBU1)*

From these findings, the general proposition can be derived:

Proposition 5: Relatively high service complexity is positively associated with the sophistication of CPA practices.

### 4.4 Balancing profitable growth and customer intimacy

Compared to the complexity of a customer relationship, CPA is a rather simplistic calculative practice that might lead to overemphasizing past profitability. Managers in this study did not follow the goal of profitability to an extent that it would have led to goal-mean-displacement. They strategized in several ways to balance *profitability* with *growth* and *customer intimacy*. First, they put results from the historic CPA in the context future profitability (cf. section 4.2.2). They mobilized calculative CPA information in a procedural way for profitability decisions, and the related to non-financial information for interactive strategizing. The latter influenced more tactical and strategic practices, such as building relationships with customers:

*“Customer profitability means a lot of things to me*. *First of all*, *it is connected to how our company handles pricing with the customers*. *[…] Secondly*, *it is connected to the business model of our company*. *I especially refer to the general analysis of the market situation outside the company*.*”(Business Unit Controller*, *SBU4)*

Second, managers mobilized CPA for strategizing by complementing it with non-financial information in the CPA reports:

*“Let’s not look at the CPA as an analysis in itself*, *but at the strategic objectives of such an analysis: these are mainly managing our customer portfolio and employing our sales force in an efficient manner*. *For achieving those goals*, *we are using other means than a pure CPA*. *For instance*, *we track the targets of the sales force*, *both at the product level and the sales plans for our top 10 customers*.*” (Director Controlling*, *SBU2)*

Some non-financial information was not formally documented, e.g., customer bargaining power, customer satisfaction, and customer proximity. Managers were concerned about a valid definition and measurement of these complex constructs, and did hence not want to include them in the formal CPA reports. They found it most appropriate if such factors entered discussions on an ad hoc basis. A management accountant emphasized how important he found such informal knowledge. He highlighted how front-line employees accounted for knowledge that unsophisticated CPA-practices did not formally record, such as unfavorable order patterns:

*“However—for many of the sales people—a lot is based on their gut feeling and their relationship with the customer*. *They can tell if a customer is profitable based on the manner he places orders over the phone*, *and so on*.*” (Business Unit Controller*, *CCU)*

Third, the authors observed that all units delegated strategizing to front-line employees (*localization*). This helped specifically the units that employed only the most basic calculative practices. The authors encountered this strategy-oriented mindset at lower hierarchical levels. Regional sales staff strategized both *procedurally* and *interactively* on CPA, and overruled low profitability in favor of customer intimacy:

*“Having a customer with low margin does not necessarily mean that we have to get rid of him immediately*. *Sometime it makes sense to keep such customers*. *[…] The low-margin customer we might want to cut might be the reason why we have 50% market share*. *Giving this customer to the competition can’t be a good idea*. *Plus*, *we can claim to be market leader in that technology*.*” (Regional Key Account Manager*, *SBU1)*

Managers rationalized why units could perform equally irrespective of CPA-sophistication. Local staff was encouraged to strategize interactively, instead of being bound to rigid policies as suggested by the external consultants:

*“The aim [of implementing CPA] was to give responsibility to our marketing and sales people*. *We want to empower them with their share of information*, *so they can make decisions based on it*. *Giving them this tool [CPA] should help them to take responsibility*.*”(Director Controlling*, *SBU5)*

*“All the marketing and sales people should be looking into more detail on this*, *because they are the ones who deal directly with customers*. *[…] The centralized controlling department [of SBU1] doesn’t need to know specifics in terms of sales and purchasing behavior and so on*.*” (Director Controlling*, *SBU1)*

From these findings, these general propositions can be derived:

Proposition 6a: The effectiveness of sophisticated CPA practices increases with the use of non-financial, strategic information.

Proposition 6b: The effectiveness of sophisticated CPA practices increases with empowerment of managers.

Proposition 6c: The effectiveness of sophisticated CPA practices increases with localization of the practice.

Proposition 6d: When factors conflict that are related to the effectiveness of sophisticated CPA practices, those factors should prevail that are most closely linked to the attainment of balanced strategic objectives.

## 5 Concluding discussion

This study responds to the call for more research on decision making with calculative practices and strategizing [5, 6]. The authors explain variations in calculative practices (CPA) that arise from strategizing in six contextually different units of a global manufacturing company. The propositions derived from the findings suggest that the sophistication of CPA practices increases with unevenly distributed customer volume, as well as with relatively high, controllable customer-specific overhead, customer-to-customer interaction, and service complexity. The sophistication of merely cost-focused CPA practices decreases with relatively diverse strategic goals. Their effectiveness generally increases with use of non-financial information, employee empowerment, and strategic alignment [28–30]. This study adds to the literature by illustrating how managers use decision-making beyond the numerical limits of calculative practices, and mobilize their practical intelligence to balance multiple strategic objectives [6].

### 5.1 Comparison of results to previous evidence

The additional insights gained in this study align at large with more basic insights from the studies reviewed in section 2. Jørgensen and Messner [18] also highlight the importance of mobilizing different strategic objectives beyond accounting information. Likewise, findings corresponds to the ideas of Nielsen et al. [24] and Perna et al. [19] to connect social-material and monetary information for decision making. Just as Sundquist et al. [20], the authors of this study see the problem of quantifying individual information on the customers as a reason why non-financial information should enter decision making in an unfiltered manner. This study confirms Ezzamel and Willmott [7] as well as Seal and Mattimoe [12], who also documented that accountants increase their strategic thinking when provided with or asked to deal with non-financial information that relates to the strategic goals of a company. The findings of this study indicate that strategizing with non-financial CPA information is more relevant than making the quantitative information from CPA an “*obligatory point of passage*” as reported by Whittle and Mueller [4]. In line with Keränen and Liozu [21], but opposed to Fish et al. [22], this study promotes the idea that the ways CPA practices are implemented should differ according to the organizational setting where they are applied. The view of this study view is strengthened when considering that overly standardized CPA practices may fail in the implementation phase, such as in van der Steen and Tillema [23]. While Balboni and Terho [25] and Wouters and Kirchberger [8] focused more on the revenue-related aspects of customer profitability, this study adds deeper insights to the cost-related aspects. Overall, CPA practices do affect performance of a company, as also suggested by Güldenpfenning et al.’s [13].

These findings underline the dynamic nature of CPA-practices within CIG. While the overarching strategic objectives remained constant, the units’ ability to contextualize their practices showcased the importance of flexibility in strategic decision-making. The study’s insights offer valuable lessons for companies seeking to align their CPA-practices with their strategic goals and optimize customer profitability. It is worth noting that one effective strategy for reducing total costs is by decreasing the unit variable cost, which leads to an increase in profitability, provided other variables remain constant. Additionally, strategic improvements can be achieved through the development of a new business plan, which can further enhance profitability and guide decision-making in a dynamic business environment.

### 5.2 Contributions to theory

Our results carry several implications for strategizing with calculative practices at the levels of praxis, practitioners, and the practice itself. First, this study helps to understand an episode of *praxis* where calculative practices gained legitimacy because managers reframed them. The process in this study resembles the three steps of “*reframing*” suggested by Jarzabkowski [16, p. 164]: In a first step, practitioners countered the consultants’ generic-action CPA. They understood CPA’s “*limits of calculability*” [18, p. 184], and anticipated goals-means-displacements (procedural strategizing). Specifically, accounting-trained managers would not accept that the original CPA gained a structural legitimacy where short-term profitability would overpower the strategic goal of customer intimacy [23]. This reframing was partly possible because top management valued diversity and wanted its managers to strategize with suggested practices (*localization*) [21]. In a second step, managers strategized interactively and reinterpreted the practice, so it could address multiple strategic objectives [20]. In a third step, the reframed practice gained both structural and interactive legitimacy, and managers actively stabilized the practice (integrative strategizing). In the reframed versions, CPA did not have if-then policies to eliminate service to customers. It became a legitimate practice in all units, because it offered managers possibilities for *alternative* ABM-initiatives instead [also cf. 24].

Second, this study helps to understand another episode of *praxis* where calculative practices were *stabilized* [1]. Managers upheld several principles that ensured CPA’s ongoing legitimacy. One principle was that CPA did not become an “*obligatory passage point*” to assess customer relationships [6, p. 316]. This is opposite to observations where calculative practices have vetoing power [24], are contested [10], manipulated, or bypassed [4, 22]. For instance, there was no obligation that tracking order patterns had to result in a detailed translation into monetary amounts for an order. Yet, managers mobilized these order patterns as a reminder of profitability that linked to the strategic objectives. CPA possessed high interpretive viability and framed strategizing through a general understanding of customer relationships, not through rigid rules [11]. A second principle was to complement the calculative practices (procedural strategizing) with non-financial information (interactive strategizing) that was explicitly stated in the CPA reports, e.g., order patterns, or implicitly entered the discourse on the CPA reports, e.g., customer bargaining power [24]. This acknowledged the limitations of the purely financial, periodic view of CPA [also cf. 20]. Non-financial information also helped to balance the short-term (*profitability*) with the long-term goals of CIG (*growth*), as well as these financial with the strategic goals (*customer intimacy*). Managers in this study contextualized CPA-practices on a case-to-case basis. They achieved an appropriate enactment of multiple strategic objectives [3]. Similar to the case study of Woods, Taylor [31], the authors find that managers used a calculative practice to focus on profitability, while simultaneously considering customer-related strategic objectives. This study enhances a general understanding, why and how quantitative customer measures are used less in informal, interpersonal communication [11]. Interactive strategizing took the form of personal interactions, where managers discussed CPA numbers. CPA was then often overruled by relevant other strategic indicators such as customer size or customer-to-customer interactions. There were no specified levels of customer profitability. As a result, CPA became subject to interpretation and negotiations among managers [8, 11, 17]. Discussions supported managers in aligning their operative decisions with strategic objectives [1]. This interactive strategizing helped to interpret strategic goals of the units and adjust the business model over time [1, 3]. The changes to the business model and the products of some units offers similarities to studies claiming that accounting discourse influences strategic positioning [2], growth [3], strategic outsourcing decisions [20, 24], or a shift from push toward pull marketing [8]. It is also in line with Jørgensen and Messner [18] who show that calculative practices can be mobilized together with strategic objectives. A third principle was that CIG’s managers admitted to the information gaps they faced. They responded with localizing CPA even further to front line employees [21]. This is consistent with Jørgensen and Messner’s [18] study, where the absence of defined accounting practices allowed R&D engineers to strategize. The authors suggest that accounting practices are predestined for localization since their numerical nature allows an inter-subjective, explicit format. This format makes it easier for staff to draw upon the structural legitimacy of numbers in communications with superiors to gain interpretive legitimacy for their activities [2]. In addition, these calculative practices are relatively simple and hence understandable for managers without specific training in accounting. This extends previous research on the use of calculative practices by staff [11, 17].

Third, this study sheds light on the roles of *practitioners* strategizing with calculative practices. The authors observed that the accounting/finance-innate strategic goal of *profitability* had to balance with strategic goals that are rather akin to strategy and management (*growth*), and marketing and sales (*customer intimacy*). It is remarkable that most of the collaboration partners had an educative affiliation with accounting, but strongly mobilized arguments related to strategy and marketing when overruling negative CPA assessments [cf. 7]. Like Jørgensen and Messner [18] and Güldenpfenning et al. [13], the authors do not encounter political conflicts between marketing (customer) and accounting (profitability)-related strategizing. The accountants were not “*policemen*” that exploited CPA to discipline colleagues from strategy, marketing, and sales [12]. Rather, managers drew upon both calculative practices as well as the related non-financial knowledge [11, 17, 20].

Fourth, the authors propose a classification of CPA as a *practice* (type I-III) and propositions for future research. Specifically Holm et al. [32] have pointed out in their field study that a lack of such framework has so far complicated large empirical studies on CPA at the population level. The authors identify a—sometimes ambiguous—pattern that sophisticated CPA-practices emerged where customer portfolios contain large, interlinked, and complex customers (marketing perspective) that consume relatively large amounts of avoidable overheads (accounting perspective). The fact that CIG employed a calculative practice that focused on customers was—most likely—responsible for the observations that managers focused on topics such as pricing and profits instead of, typically, budgets and costs [25]. In the same vein, the authors find it sensible that managers felt nudged to mobilize arguments from marketing and strategy—instead of research and engineering if product profitability had been the focus. Thereby, CPA established formal structures that forced managers to think about profitability, what drove it, and which of the relevant profitability drivers were neglected [2]. The study’s findings link to studies that found relations between customer accounting and competitive market orientation [3], cost structures [17], customer relationships [11], and the realized strategy [2]. The authors do not propose optimal calibrations of CPA-practices and their context as contingency theory would. The authors rather intend to illuminate how multiple strategic objectives create variation in accounting practices [18, 24], and how these practices then again support strategizing once they gained legitimacy [2, 4].

### 5.3 Implications for practice

First, the fact that CIG’s top management let the calculative practice develop locally helped embedding CPA and avoided resistance. CPA is a crude reflection of both the profitability and the strategic importance of a customer. Yet, the managers did not fundamentally reject CPA, because they were not forced to use it as the ultimate benchmark for customer relationships [6]. They rather appreciated the (limited) possibilities of this calculative practice [similar: 2]. Thereby, CPA became a starting point for informed reflections on the role of the customer for reaching strategic objectives [18, 24, 33]. The authors also add these insights on customer accounting in a B2B setting, which is often relatively complex and still under-researched.

Second, this study critically assesses the unthoughtful pursuit of fashionable, highly sophisticated *best-practices* [34]. The consultants promoted a generic CPA that would have ignored the multi-dimensionality of strategic objectives. Managers feared that structurally embedding such a CPA could have led to means-goals-displacements [1]. While some units accepted some of the consultants’ suggested practices, others adopted a reduced version. And all units complemented the practice beyond its calculative approach [11, 17, 18]. Even the units with simple CPA-practices initiated situated managerial action (ABM) when the CPA indicated overly negative outcomes. Hence, the authors studied a process of first reframing and then stabilizing adapted CPA-practices, not cases of resistance or failed implementations [1]. This case is an encouragement for managers make sense of fashionable practices in their strategic context, instead of blindly following ‘best practices’ and generic ‘if-then’ activities.

### 5.4 Limitations and future research

Our findings are subject to several limitations that raise issues for future research. First, even calculative practices such as CPA contain much more than the financial output of the ERP system. Hence, they are fuzzy to delimit. This study cannot give a full account of CPA at a company as large as CIG, and the authors’ writings can only reflect (representative) episodes of praxis.

Second, calculative practices may be transferable across organizations, but they will still develop differently according to other cultures, strategic objectives, and industries [3]. Future researchers could broaden their focus beyond CPA and study such practices in different contexts. Researchers could look at further contextual factors, such as uncertainty [11, 35], management characteristics [9], or linguistic framing of practices [34].

Third, the authors only look at strategic applications to CPA and mainly interview high-ranking and middle managers that are often associated with accounting. This is the case because they had the authority to administer CPA in this company, and the authors intended to investigate the top-level *strategic* applications. Future studies could shed more light on the various ways front-line staff interacts with customers on an operative level.

Fourth, the authors only investigated the praxis of stabilizing the practice over several months, while the authors only heard reports about the reframing of the practice. There could be an ownership bias evident in the key informants, who convey to the authors the ex-post rationalization of why they reframed and stabilized CPA in a way that they initially might not have intended. Future studies could look at further episodes of praxis, such as the early emergence of CPA in a company setting, and analyze the actual meetings and argumentations of managers while practices are still being customized [3]. Last, future research could take a closer look at the supply-side of practices and understand the motivation of external consultants in recommending full implementations [1].

## Supporting information

S1 Dataset (DOCX)
