# Supplementary material for: Customer Profitability Analysis in decision-making–The roles of customer characteristics, cost structures, and strategizing
Source: PLoS One. 2024 May 22;19(5):e0296974. doi: 10.1371/journal.pone.0296974 (PMC11111084; doi:10.1371/journal.pone.0296974)
Supplement: S1 Dataset — (DOCX) [file pone.0296974.s001.docx]

Customer profitability analysis
in decision-making –
The roles of customer characteristics,
cost structures, and strategizing

# Minimal dataset

**The minimal dataset of this study is the interview guideline listed in this appendix:**

A thorough approach was implemented to guarantee the relevance and usefulness of the interview questions for this study on CPA in manufacturing. To fully comprehend the important aspects of CPA, the research team build on a literature review that was initially more extensive than what is presented in this article. Based on this framework, the team members had a number of brainstorming meetings and discussions to produce an initial collection of questions. With the unique organizational context in mind, questions were improved and put into a logical format. The pilot study among peers provided the research team the chance to identify any ambiguities or areas where the questions’ language, arrangement, or substance may be improved. After carefully examining the feedback from the pilot research participants, questions were revised. The research team then examined and approved the final set of questions to make sure they adequately addressed the study’s goals and captured the key information required to understand CPA as its whole within the study’s context.

**Guideline list of questions for the semi-structured interviewees**

1. How would you define the concepts of “customer profitability” and “customer profitability analysis” (CPA) in the context of your business?

2. What specific outcomes do you anticipate from implementing CPA within your organization?

3. Can you provide an overview of the current content and components of your CPA? What specific analyses or metrics are currently employed?

4. In your opinion, how would you assess the quality of the information generated through your current CPA processes?

5. Could you elaborate on the roles and responsibilities of individuals involved in the CPA within your organization? How do they contribute to the overall process?

6. Are there any additional analyses or types of information that you believe would be valuable to incorporate into your CPA practices?

7. How do you leverage the insights and findings derived from your CPA? How do they influence decision-making and control within your organization?

8. What are the main challenges or limitations you have encountered with regard to CPA? If you had the opportunity, what aspects or elements would you modify or improve?

9. How would you rate your overall level of satisfaction with the current implementation and execution of CPA in your organization? Are there any specific areas where you feel more or less satisfied?

The full replies to the questions, as well as all other gathered data at the company site, may not be published due to participant privacy and ownership of these data by third parties.
